# Determinants of continuum of care for maternal, newborn, and child health services in rural Khammouane, Lao PDR

**DOI:** 10.1371/journal.pone.0215635

**Published:** 2019-04-23

**Authors:** Saki Sakuma, Junko Yasuoka, Khampheng Phongluxa, Masamine Jimba

**Affiliations:** 1 Department of Community and Global Health, Graduate School of Medicine, The University of Tokyo, Tokyo, Japan; 2 Department of International Affairs and Tropical Medicine, Tokyo Women’s Medical University, Tokyo, Japan; 3 Research and Education Center for Prevention of Global Infectious Diseases of Animals, Tokyo University of Agriculture and Technology, Fuchu City, Tokyo, Japan; 4 National Institute of Public Health, Ministry of Health, Vientiane, Lao PDR; Seoul National University College of Medicine, REPUBLIC OF KOREA

## Abstract

**Introduction:**

The concept of continuum of care has gained attention as measures to improve maternal, newborn, and child health. However, little is known about the factors associated with the coverage level of continuum of care in Lao PDR. Therefore, this study was conducted 1) to investigate the coverage level of continuum of care and 2) to identify barriers and promoting factors that are associated with mothers’ continuation in receiving services in rural Lao PDR.

**Methods:**

A community-based, cross sectional study was conducted in a rural district in Khammouane Province, Lao PDR, using a structured questionnaire. The outcome to the express continuum of care was assessed by the modified composite coverage index (CCI) that reflects ten maternal and child health services.

**Results:**

In total, 263 mothers were included in the final analyses. Only 6.8% of mothers continued to receive all MNCH services. Five factors were shown to have statistically significant associations with modified CCI score: higher educational attainment (B = 0.070, p<0.001), being a farmer (B = -0.078, p = 0.003), receiving the first antenatal care within the first trimester (B = 0.109, p<0.001), longer distance from district hospital (B = -0.012, p<0.001), and discussion with husband or family members (B = 0.057, p = 0.022).

**Conclusions:**

In this study, we introduced the modified CCI to better explain the utilization of preventive maternal and child health services along with the continuum of care. By utilizing the modified CCI, we identified five factors as determinants of continuum of care. Furthermore, new and modifiable promoting factors were identified for continuum of care: receiving the first antenatal care within the first trimester and family and male involvement. Such demand side actions should be encouraged to improve the continuity of MNCH service use.

## Introduction

Maternal, newborn, and child health (MNCH) remains one of the main global health issues. Approximately 303,000 women still succumbed to complications of pregnancy and childbirth in 2015, and about 99% of such deaths occurred in low- and middle-income countries [1]. In addition, in 2015, 5.9 million deaths occurred for children under the age of five and 2.7 million deaths during the neonatal period [2]. The high number of these mortalities in some areas of the world reflects inequities in access to health services [3].

To improve MNCH through integrated service delivery, the concept of continuum of care has been highlighted [2,4–6]. The term “continuum of care” for MNCH has two important components: 1) time component referring to the continuity of care starting from pre-pregnancy, pregnancy, childbirth, postpartum, and childhood, and 2) place component referring to the level of facility at which care is taking place [7]. Interventions and strategies to strengthen the continuum of care have recently been discussed in MNCH [8–10].

Lao People’s Democratic Republic (Lao PDR) is a land-locked country in Southeast Asia categorized as a lower-middle income country. Of 6.5 million people, 67.1% were estimated to live in rural mountainous areas and 7.9% in areas with no roads in 2015 [11]. To reduce maternal, neonatal, and child mortality, the government of Lao PDR has initiated a number of strategies in primary healthcare settings in collaboration with international partners. In 2007 the Ministry of Health established a Maternal and Child Health- Expanded Program for Immunization Technical Working Group and developed the Strategy for the Integrated Package of Maternal, Neonatal and Child Health Services 2009–2015 [12]. An essential package has been clarified in the strategy with regards to the continuum of care [12,13].

Despite these efforts, Lao PDR faces significant disparities in MNCH services coverage among urban and rural residents and also within the urban and rural residents [6,14]. Factors associated with individual MNCH service have been studied for antenatal care (ANC), delivery place, and vaccinations [14–18]. However, factors associated with the level of continuity of MNCH services and postnatal care (PNC) are still unknown [18]. Success to improve the continuum relies on a better understanding of the care-seeking behaviors and factors contributing to the disparities [19].

In the current study, the objectives were 1) to investigate the coverage level of continuum of care, in addition to looking at MNCH services individually among mothers, and 2) to identify barriers and promoting factors that are associated with mothers’ continuation in receiving services in rural Lao PDR. Furthermore, we sought to identify modifiable factors which mothers can contribute by themselves to improve the continuum of care.

## Methods

### Sampling methods

#### Study design and setting

This is a community-based, cross sectional study, which was conducted in Xaybouathong district in Khammouane province, Lao PDR. Khammouane province is a predominantly rural area in central Lao PDR where forested mountain terrain is located [20]. Xaybouathong district was selected for the study due to the low coverage of MNCH services. Facility-based delivery coverage, for example, was lower compared to the entire province (31.6%) [21]. The district hospital catchment area out of six subdivision catchment areas in Xaybouathong district was purposefully selected for this study to allow for adequate accessibility by car during a rainy season. Within the catchment area, 9,978 people (1,767 households) live in 22 villages, and the number of births was 416 in 2014 [21]. To avoid undue influences on interview data, an area that has not been previously used for a study or conducted interventions concerning MNCH by external organization within past ten years was chosen.

### Participants

Inclusion criteria for the participants were 1) mothers aged from 16–49 years old who had children between 8 to 23 months old at the time of interview and 2) residence in the selected district since the last child’s pregnancy. Mothers who have severe physical and mental problem and difficulty participating in the study were excluded.

Power calculation for mothers who have a child aged 8 to 23 months was performed using Open Epi version 3 resulting in a minimum sample size of 254. This was based on the assumption that educational attainment is significantly associated with antenatal care (ANC) coverage (Primary education: 56.1%, Lower secondary education: 72.7% according to Lao Social Indicator Survey 2011–2012) [22]. Two-sided confidence level was set at 95% and power at 80%. In total, 279 eligible mothers were recruited.

### Data collection

Data collection was carried out from August to September, 2016. Face-to-face interviews were conducted at either each mother’s house or community space using a structured questionnaire. The questionnaire was developed based on the Lao Social Indicator Survey 2011–2012 questionnaire [23] and measurement tools used in previous studies [22,24]. The questionnaire was initially prepared in English and translated into Lao language and then translated back to English. The structured questionnaire consisted of four sections: 1) socio-demographic and household characteristics including age, education, occupation, ethnicity, religion, marital status, children/parity, family income, and spouses’ characteristics, 2) history of pregnancy and delivery, 3) MNCH services utilization, and 4) knowledge about MNCH services.

Six research assistants were recruited to conduct face-to-face interviews. Before the data collection, a one-day training program was conducted twice for their acquiring interviewing techniques and understanding the objectives clearly by comprehending each question. A pre-test was conducted in a village within the same district hospital catchment area selected by the district health office head.

### Modeling

#### Dependent variables

Two basic approaches are widely used for measuring MNCH intervention coverage. First is to look at separate analyses for each effective MNCH intervention. Therefore, utilization of the MNCH services was measured by asking whether each mother had received health services during pregnancy, childbirth, postpartum, and childhood periods. Ten outcomes on the utilization in MNCH services were used. These are measured mainly according to the World Health Organization (WHO) definitions. ANC was defined as care that pregnant women received from skilled healthcare providers; and its number was considered in this study. Neonatal tetanus protection was defined when either women received tetanus toxoid injection during pregnancy or when health provider considered them to be protected. Facility-based delivery was defined as the delivery of pregnant women at a health facility. Skilled birth attendant (SBA) delivery was defined as delivery attended by healthcare professionals (doctor, midwives, and nurses). PNC was defined when women or newborn received a health check within two days of delivery. Children’s immunization coverage was measured from either vaccination records in the immunization cards or from the immunization logbook. Immunization was defined when a child received each vaccination previously. Family planning was defined as use of contraception for currently married or in union who are using a contraceptive method, and excluded currently pregnant mothers. All services were coded ‘yes/no’.

Second, in addition to each service analysis, the composite coverage index (CCI) was used to calculate the mothers’ continuity utilization of local MNCH services. CCI is a weighted score reflecting coverage of eight reproductive, maternal, newborn and child health interventions along with the continuum [25]. CCI has been used as an effective way to summarize and show health inequality to compare MNCH interventions’ coverage between countries and within countries over time.

Since no representative measurement exists to express the coverage level of continuum care at individual level, a modified CCI was created to evaluate the MNCH services’ utilization. Modified CCI was specifically designed for preventive MNCH interventions. The equation for modified CCI places equal weight to cares for pregnancy, childbirth, and postnatal, as well as services for immunizations and family planning. Management for sick children was removed and other preventive interventions were added to the original CCI. Maternal care includes four or more visits to ANC (ANC) and neonatal tetanus protection (TTN); childbirth includes facility-based delivery (FD) and SBA; PNC includes for mother and newborn (PNCM and PMCN); immunization includes BCG, three doses of pentavalent vaccine (Penta;DPT-HepB-HIB: DPT, Hep B: Hepatitis type B, HIB: Heamophilus influenza type B), and three doses of polio immunizations (Polio) at the age of 8 to 23 months; and family planning (FP) includes prevalence of contraceptive use. For each intervention use, one for yes and zero for no was entered to calculate modified CCI for all women, which range from zero to one.

$$Modified\;CCI=\frac{1}{5}\left(\frac{ANC+TTN}{2}+\frac{FD+SBA}{2}+\frac{PNCM+PNCN}{2}+\frac{BCG+Penta+Polio}{3}+FP\right)$$

Another way of explaining the continuum of care was compared in the study by adding all ten MNCH services used in the modified CCI as yes/no. The Spearman’s rank coefficient showed 0.97 and the linear regression showed almost the same result. Therefore, the index is useful to evaluate mothers’ continuum of care for MNCH service use.

#### Independent variables

Based on the Owilli’s continuum of care framework, conceptual model was divided into four major components of family and individual, community, socio-economic status, and baby’s characteristics conceptual model [26]. Family and individual factors included mother’s age, spouse’s characteristic, marital status, ethnicity, religion, and household information. In addition, knowledge about each service, whether mother had discussed about the services with husband or other family member, and who provided the most important information were also obtained. Community characteristic factors included rural with/without road and the distance to health facility. Socio-economic status factors included mother’s education attainments, husband’s education attainments, mother’s occupation, and family income. Baby’s characteristic factor included child age, sex, and child order.

### Data analysis

All data were first coded and entered into Epi Data version 3.1 and then transferred to Stata version 13 for statistical analyses. Descriptive analysis (number, frequency, and percentage) was used to summarize the characteristics of each variable. Either Chi square test or Fisher’s exact test was used to examine the association between each MNCH service and related variables. Then, multiple logistic regression analysis was performed to examine the association between each MNCH service and independent variables, adjusted for possible confounders. Furthermore, the associations between modified CCI and independent variables were checked by multiple linear regression analysis. Mean was used as cutoff for household size. Median was used as cutoff for age and distance from the hospital.

For multiple linear regression analysis, independent variables and all the other possible factors that could influence the service use from previous studies were put into the first model. Correlations coefficients and the variance inflation factor were checked. Backward elimination was used to reach to the final model with five factors. P-value of <0.05 was considered statistically significant.

#### Ethical considerations

This study was approved by the Research Ethics Committee of the Graduate School of Medicine, the University of Tokyo, Japan. The same proposal and other necessary documents were approved by the National Ethics Committee for Health Research, the Ministry of Health Lao PDR. All participation was voluntary, and written informed consent was obtained at the beginning of the interview.

## Results

### Socio-demographic characteristics

In total, there were 263 mothers included in the analyses. The socio-demographic characteristics of the participants are shown in Table 1. The average number of household members was 6.2 (standard deviation (SD) 2.5). The average age of mothers and husbands were 26.3 years (SD 6.1) and 30.8years (SD 7.3), respectively. Ethnicity and religion of the mothers were Lao-Tai 226 (85.9%) and Buddhist 180 (68.4%). The majority of mothers (79.9%) and their husbands (63.5%) had either no education or up to primary school education and worked as farmers (68.1%). About a quarter (25.1%) of children were 8 to 11 months and about half (49.8%) of the children were boys. On average, mothers lived 6.6 km (SD 4.2) away from the district hospital. The average number of their past pregnancies was 3.2 (SD 2.4).

**Table 1:** Socio-demographic characteristics of participants (n = 263).

Variables	Mean (SD)	n	%
**Household size**	6.2 (2.5)		
**Mother's age (years)**	26.3 (6.1)		
**Husband's age (years)**	30.8 (7.3)		
**Number of children**	2.9 (2.0)		
**Number of pregnancies**	3.2 (2.4)		
**Marital status**			
Married		222	84.4
Others		41	15.6
**Mother's educational attainment**			
No education		93	35.4
Primary		117	44.5
Secondary or higher		53	20.2
**Mother's occupation**			
Farmer		179	68.1
Others		84	31.9
**Husband's educational attainment**			
No education		51	19.4
Primary		116	44.1
Secondary or higher		96	36.5
**Ethnicity**			
Lao/Tai		226	85.9
Others		37	14.1
**Religion**			
Buddhist		180	68.4
Others		83	31.6
**Child age (months)**			
8–11		66	25.1
12–23		197	74.9
**Sex of child**			
Boy		131	49.8
Girl		132	50.2
**Distance to district hospital (km)**	6.5 (4.2)		

SD: standard deviation, km: kilometer

### Utilization of MNCH services

MNCH service utilization is shown in Table 2 (see also S1 Fig). Among all, 182 (69.2%) received at least one ANC, 143 (78.6%) received four times or more ANC, and 108 (41.1%) received their first ANC within the gestational age of first trimester (up to 12 weeks). Among 263 mothers, 239 (90.9%) received neonatal tetanus protection. In addition, among 263 mothers, only 18 (6.8%) continued to receive all 10 services used in modified CCI which include ANC 4 or more visits, neonatal tetanus protection, facility-based delivery, delivery assisted by SBA, PNC for mother and newborn, BCG, Penta, Polio, and FP.

**Table 2:** Coverage of MNCH services (n = 263).

Variables	N	%
**Antenatal care(at least 1 visit)**		
Yes	182	69.2
No	81	20.8
**Antenatal care(4 or more visits)**		
Yes	143	54.4
No	120	45.6
**First antenatal care visit**		
≦12 weeks	108	41.1
>12 weeks	155	58.9
**Neonatal tetanus protection**		
Yes	239	90.9
No	24	9.1
**Delivery place**		
Facility	78	29.7
Home	185	70.3
**Delivery assistant**		
SBA	80	30.4
TBA/VHV	14	5.3
Husband	113	43.0
Alone	10	3.8
Others	46	17.5
**Postnatal care for mother**		
Yes	81	30.8
No	182	69.2
**Newborn care**		
Yes	84	31.9
No	179	68.1
**Childhood immunization**		
BCG	255	97.0
HepB0	105	39.9
Penta3	262	99.6
Polio3	262	99.6
Measles	214	81.4
Japanese Encephalitis	163	62.0
**Family planning**		
Yes	112	42.6
No	151	57.4
All 10 services*		
Yes	18	6.8

SBA: skilled birth attendant, TBA: traditional birth assistant, VHV: village head volunteer,

HepB0: hepatitis type B vaccine birthdose, BCG: Bacillus Calmette-Guerin, Penta: pentavalent vaccine (DPT-Hepatitis B -haemophilus influenza type B, DPT: diphtheria-pertussis-tetanus

*:All 10 services include: ANC 4 or more visits, neonatal tetanus protection, facility-based delivery, delivery assisted by SBA, PNC for mother and newborn, BCG, Penta, Polio, and FP

Of 263, 78 mothers (29.7%) delivered in a medical facility including central-level to district-level hospitals and health centers. Among all, 80 (30.4%) were assisted by a SBA, 14 (5.3%) by TBA or village health volunteer (VHV), 113 (43.0%) by husband, 10 (3.8%) delivered alone, and 46 (17.5%) by others.

About a third (30.8%) received PNC for herself and 84 (31.9%) received PNC for newborn. Of 78 women who delivered in facility, 62 (79.5%) received both PNC services for mother and newborn. On the contrary, of the 185 mothers who delivered at home, only 10 (5.4%) received PNC.

All children received at least one vaccination. Its coverage differed by vaccination: 97.0% for BCG, 39.9% for Hepatitis B at birth, 99.6% for three doses of pentavalent and polio, 81.4% for measles, and 62.0% for Japanese encephalitis.

### Determinants of continuum of care

Modified CCI was calculated for each mother and used as an indicator of continuum of care. Average score for modified CCI was 0.55 (SD 0.22) (S2 Fig for Histogram of modified CCI). A multiple linear regression model was run to examine the determinants of modified CCI (Table 3) (See also S1 Table for Results of multiple regression for each MNCH service). Five factors were shown to have statistically significant associations: higher educational attainment (beta = 0.226, p<0.001), being a farmer (beta = −0.162, p = 0.003), receiving the first ANC within the first trimester (beta = 0.239, p<0.001), longer distance from district hospital (beta = −0.233, p<0.001), and discussion with husband or family members (beta = 0.116, p = 0.022). All raw research data and questionnaire are shown in S1 Dataset and S1 Questionnaire.

**Table 3:** Multiple linear regression: Factors associated with modified CCI (n = 263) (Adjusted R^2^ = 0.378).

Variables	Beta	SE	T	p-value
**Mother’s educational attainment**	0.226	0.018	3.910	<0.001
**Mother’s occupation (farmer)**	−0.162	0.026	−3.020	0.003
**Months of first ANC visit (1**^**st**^ **trimester)**	0.239	0.024	4.630	<0.001
**Distance to district hospital (km)**	−0.233	0.003	−4.210	<0.001
**Discussed with husband or family**	0.116	0.025	2.300	0.022

CCI: Composite coverage index, SE: standard error, ANC: antenatal care, km: kilometer

## Discussion

To the authors’ knowledge, this is the first study to focus on the coverage level of MNCH services and investigate predictors of MNCH utilization in the form of modified CCI in Lao PDR. The study demonstrated important findings. We introduced the modified CCI to better explain the coverage level of preventive MNCH services utilization along with the continuum of care. First, only 6.8% of mothers received all ten MNCH services, highlighting the low MNCH coverage and underscoring room for improvement. Second, the study identified barriers and promoting factors involved in continuum of care. The findings indicate that receiving the first ANC within the first trimester and having discussion about the services with husband or other family members were new promoting factors for continuum of care. In addition, factors such as women’s education was positively associated, and being a farmer as occupation and distance from the health facility were negatively associated with the modified CCI score.

Although previous studies in Cambodia and Ghana identified the factors associated with continuum of care [19,27], MNCH services included only ANC, SBA, and PNC. This study adds to the current literature as the modified CCI extended the time dimension of continuum of care by adding preventive MNCH interventions. In addition, five factors associated with modified CCI were identified.

This is the first study to investigate the coverage level of continuum of care in Lao PDR. The level of continuum of care varied among mothers in rural Lao PDR. Less than 10% of them received all the MNCH services which lower than a previous study in Cambodia which varied from 14 to 24% among rural provinces, although they used a different way of assessing the continuum of care [19]. However, the low rate of receiving all MNCH services might have been influenced by the low rate of facility-based delivery and SBA. This may be attributed to the traditional belief of home delivery. The finding indicates the necessity of further study in Lao PDR to improve the coverage of MNCH services.

In this study, receiving the first ANC within the first trimester showed a positive association with receiving many MNCH services. This finding is consistent with the WHO’s ANC recommendations, which mentioned that one contact should be provided in the first trimester [10]. A possible explanation would be that mothers can capture necessary information such as iron folic acid supplementation, tetanus toxoid immunization, counseling on maternal and infant nutrition, birth and emergency preparedness, and other MNCH services. Furthermore, receiving the first ANC earlier can link to the next MNCH service use and care [19,28,29]. In South Africa, early access to ANC had an association with lower maternal deaths [30]. Thus, receiving the first ANC within the first trimester may increase the mother’s utilization in MNCH services, which may be connected to the decrease in maternal deaths.

This study demonstrated that another important factor in continuum of care was family and male involvement. In particular, male involvement was mentioned in the recommendations to promote MNCH utilization by WHO while family involvement was not emphasized [31]. In addition, in Nepal and Myanmar, the benefits of male involvement have shown significant association with MNCH utilization [32,33]. Discussing about MNCH services with family or husband was used as family and male involvement in this study. Thus, our findings are in agreement with these findings. This could be because discussion with family could support woman’s empowerment which resulted in MNCH service use. In rural Lao PDR, family and men still play a significant role in the decision-making process; this could be a possible explanation of importance of family involvement in MNCH. Our findings indicate that efforts to increase awareness and improve male involvement could lead to better MNCH utilization.

In this study, factors such as mothers’ higher education, occupation as non-farmer, shorter distance from the health facility were significantly associated with higher modified CCI score. These findings are all similar to previous studies [16,19,27,34]. To improve the level of education and access to health facilities, Lao PDR government is working toward increasing educational enrollment and increasing health facilities and outreach sessions in integrated services. Our study site was in a rural district with unpaved roads. The accessibility to health facility might have reflected the result. In addition, community health workers have shown effectiveness for delivering preventive maternal and child health interventions in low- and middle-income countries [35]. Lao PDR government may focus more on the utilization of community health workers as a resource to deliver MNCH interventions.

While education, accessibility, and occupation are factors which require long-term intervention, we highlight our findings on the modifiable factors that mothers can change by themselves in the short term. Interventions and efforts to educate the mothers on the importance of early ANC visit and family/male involvement could lead to improved MNCH in the short-term.

### Limitations

This study has several limitations. First, the children’s vaccination age is one of the limitations since whether children received the vaccination during the recommended period by the government was not considered. This may possibly overestimate the vaccination coverage. At the same time, measles vaccination was excluded from the modified CCI since children received measles vaccination from 8 to 11 months. If measles vaccination was included in modified CCI, the criteria of child’s age should be reconsidered to avoid underestimating the percentage of vaccinated children. Second, since deceased women and children were not included in the study, it may cause overestimating the utilization of MNCH services and underestimating the magnitude of the problem. Third, the interpretation of study findings is also subject to information bias. Although most mothers had yellow cards for vaccination, several answers from women were based on their memories, recall bias may have occurred. Fourth, the study was conducted in the district hospital catchment area and did not cover catchment areas of health centers. Due to this limitation, the MNCH coverage may have shown better results. Because Lao PDR has diversity in ethnicity, MNCH situation may differ according to ethnicity or languages they speak. Finally, the study only covered the demand-side factors. Supply-side factors and the quality of the services are the area of future research. Also, the modified CCI should be further validated for further research, and the relationship between MNCH service utilization and mortality should be given more focus along with the continuum of care.

## Conclusion and recommendations

In conclusion, we demonstrated a low percentage of continuity in continuum of care in rural Lao PDR, as evidenced by only 6.8% of mothers receiving all MNCH services. New promoting factors for the continuum of care were demonstrated in this study expressed by modified CCI, including receiving the first antenatal care within the first trimester and family and male involvement in MNCH. This is an important finding since they are modifiable factors which can be promoted in short term.

As a recommendation to increase MNCH services utilization, community authorities and health workers should emphasize the importance of early first ANC visit and promote family and male involvement. Furthermore, modified CCI should be studied throughout the country to find an efficient way to improve the MNCH outcomes and the equity of MNCH service utilization.

## Supporting information

S1 Fig Coverage of maternal, newborn, and child services. (PDF)

S2 Fig Histogram of modified composite coverage index. (PDF)

S1 Table Results of multiple regression for each MNCH service. (PDF)

S1 Dataset (XLXS)

S1 Questionnaire (PDF)
