# Supplementary material for: Determinants of continuum of care for maternal, newborn, and child health services in rural Khammouane, Lao PDR
Source: PLoS One. 2019 Apr 23;14(4):e0215635. doi: 10.1371/journal.pone.0215635 (PMC6478320; doi:10.1371/journal.pone.0215635)
Supplement: S1 Fig — (PDF) [file pone.0215635.s001.pdf]

**S1 Figure. Coverage of maternal, newborn, and child services (n=263)**

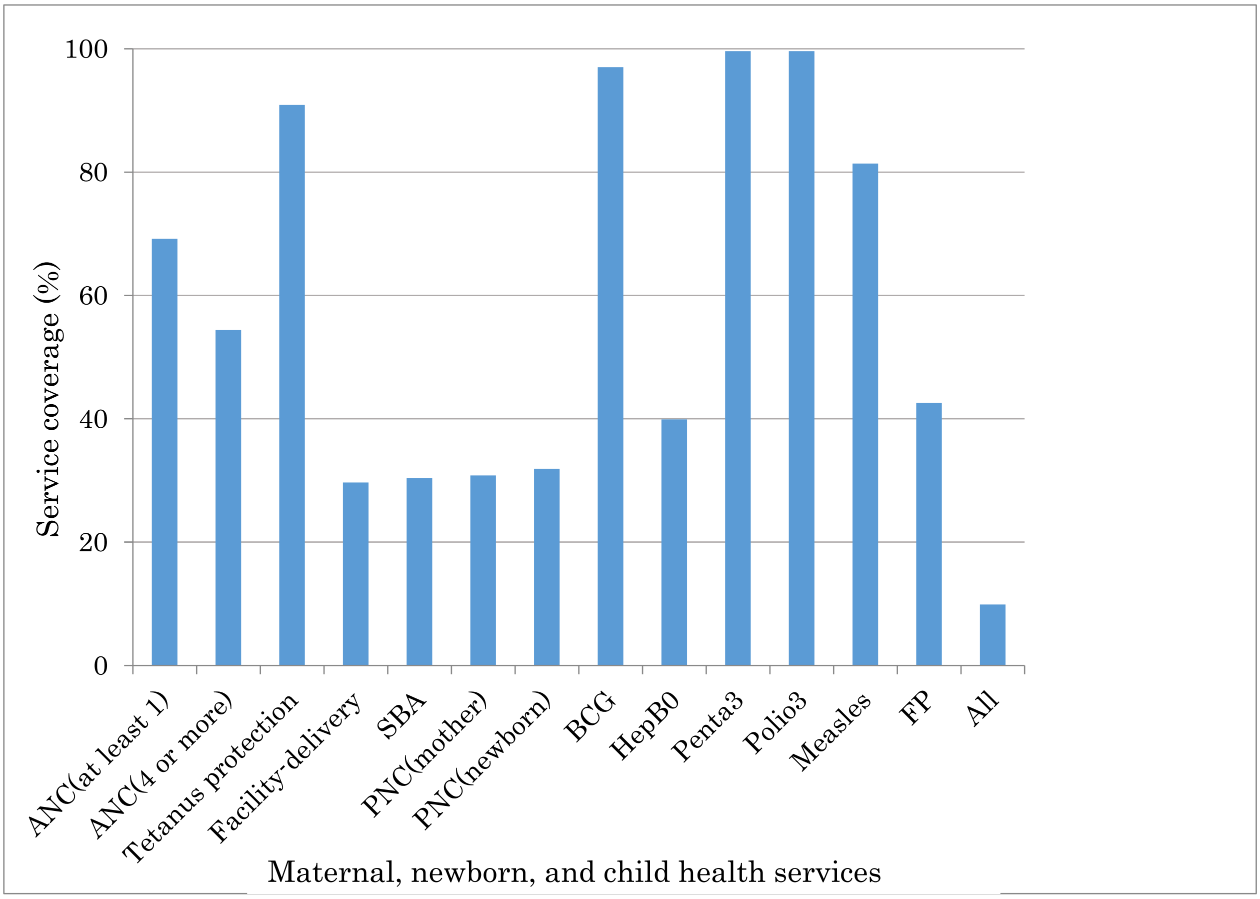


ANC: antenatal care, SBA: skilled birth attendant, PNC: postnatal care,

BCG: Bacillus Calmette-Guerin, HepB0: hepatitis type B vaccine birthdose

Penta: pentavalent vaccine (DPT-HepatitisB -haemophilus influenza tupe B, DPT: diphtheria-pertussis-tetanus

FP: family planning
