# Supplementary material for: Determinants of continuum of care for maternal, newborn, and child health services in rural Khammouane, Lao PDR
Source: PLoS One. 2019 Apr 23;14(4):e0215635. doi: 10.1371/journal.pone.0215635 (PMC6478320; doi:10.1371/journal.pone.0215635)
Supplement: S2 Fig — (PDF) [file pone.0215635.s002.pdf]

S2 Figure. Histogram of modified composite coverage index (n=263)

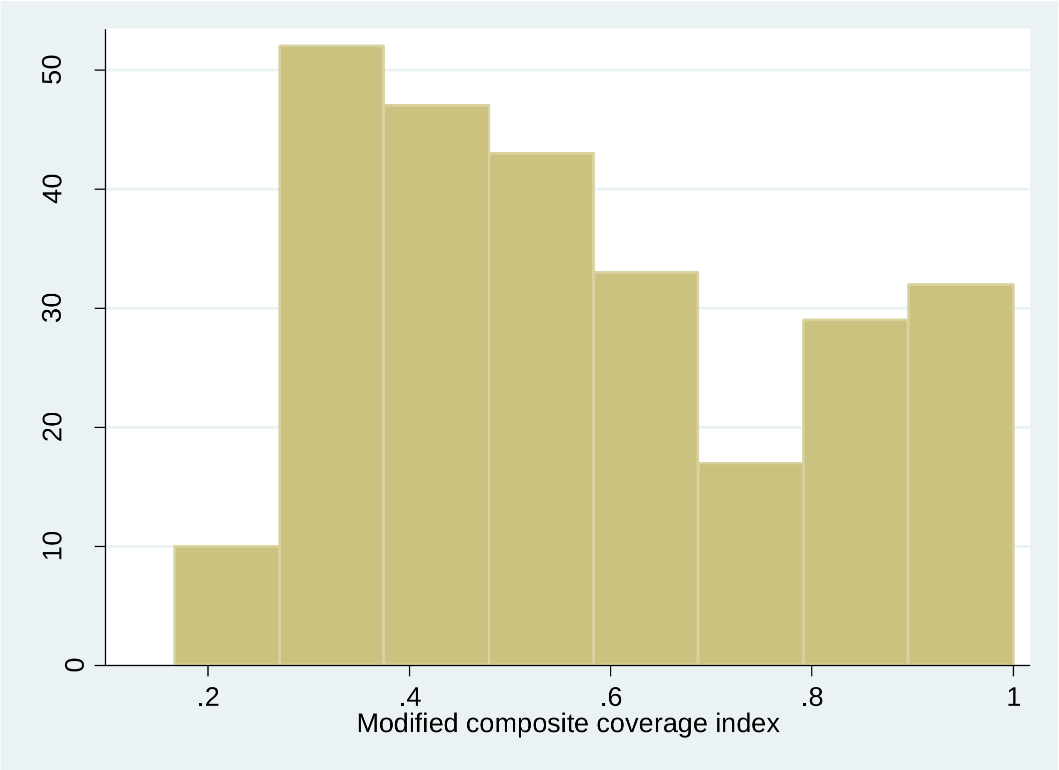

Modified composite coverage index (Mean SD): 0.55 SD 0.22

CCI: composite coverage index, SD: standard deviation
