# Supplementary material for: Determinants of continuum of care for maternal, newborn, and child health services in rural Khammouane, Lao PDR
Source: PLoS One. 2019 Apr 23;14(4):e0215635. doi: 10.1371/journal.pone.0215635 (PMC6478320; doi:10.1371/journal.pone.0215635)
Supplement: S1 Table — (PDF) [file pone.0215635.s003.pdf]

S1 Table. Results of multiple regression for each MNCH service

Variables/Services	ModifiedCCI[a]	ANC[b]	FD[b]	PNC[b]	IMM[b]	FP[b]
Household number (＞6)		↓				
Child order (First child)			↑	↑		
Mother's age (≧26 years)				↑		
Educational attainment	↑	↑	↑			
Occupation (Farmer)	↓	↓	↓	↓		
Age of first pregnancy (≦18)			↓			
Ethnicity (Lao-Tai)		↑				
Religion (Buddhist)		↓				
Distance (≧8km)	↓	↓	↓	↓		
Discussed with husband or family	↑	↑		↑		↑
Child age (12-23 months)	—	—	—	—	↑	↑
First ANC visit (≦1st trimester)	↑	—	—	—	—	—

MNCH: Maternal, newborn and child health

CCI: Composite coverage index

ANC: Antenatal care

FD: Facility delivery

PNC: Postnatal care

IMM: Immunization

FP: Family planning

[a]: Multiple linear regression model

[b]: Multiple logistic regression model

↑: positive association

↓: negative association

—: was not analyzed

blank space: not significant
