# Supplementary material for: Determinants of continuum of care for maternal, newborn, and child health services in rural Khammouane, Lao PDR
Source: PLoS One. 2019 Apr 23;14(4):e0215635. doi: 10.1371/journal.pone.0215635 (PMC6478320; doi:10.1371/journal.pone.0215635)
Supplement: S1 Questionnaire — (PDF) [file pone.0215635.s005.pdf]

S1 Questionnaire.

Continuum of Care for Maternal, Neonatal and Child Health Services in Lao PDR

QUESTIONNAIRE

Check before interviewer begins the survey:

□ Is your last child under two years old (8-23 months)?
□ Are you the mother of the child?
□ Is your age 16-49 years old?
□ Got Informed Consent?

ID of interviewer: ________________ ID of participant:_________

Date of interview: __ __ / __ __ / __ __ __ __

HOUSEHOLD INFORMATION PANEL		
A	Village Name:	
B	Area:	Rural with road…………………………………………… 1<br>Rural without road……………………….................. 2

Section I: Socio-demographic and Household Characteristics

I would like to start asking questions about your social-demographic and household characteristics.

NO.	QUESTIONS	ANSWER AND CODING CATEGORIES	SKIP
S1	Number of household member including yourself.	……………….…………………….__ __people	
S2	Number of children <15years in your household?<br>Probe: specify ages in S3	…………………………….……….__ __people	

S3. Now I would like to record all of your births, whether still alive or not, starting with the first one you had.

Record twins and triples on separate line.

No of Baby	Status of giving birth<br>Deliviered:<br>1 One/single<br>2 Twin	Sex<br>1 Boy<br>2 Girl	Date of birth<br>Day/ Month/ Year<br>(DK:99)	How old was he/her at last birthday?<br>Age<br>< 5 yr	 <br>>5 yr	Is he/she still alive?<br>1 Yes<br>2 No	Is he/she living with you?<br>1 Yes<br>2 No
1	1 2	1 2	_ _ / _ _ / _ _ _ _	__ m	__ yr	1 2	1 2
2	1 2	1 2	_ _ / _ _ / _ _ _ _	__ m	__ yr	1 2	1 2
3	1 2	1 2	_ _ / _ _ / _ _ _ _	__ m	__ yr	1 2	1 2
4	1 2	1 2	_ _ / _ _ / _ _ _ _	__ m	__ yr	1 2	1 2
5	1 2	1 2	_ _ / _ _ / _ _ _ _	__ m	__ yr	1 2	1 2
6	1 2	1 2	_ _ / _ _ / _ _ _ _	__ m	__ yr	1 2	1 2
7	1 2	1 2	_ _ / _ _ / _ _ _ _	__ m	__ yr	1 2	1 2
8	1 2	1 2	_ _ / _ _ / _ _ _ _	__ m	__ yr	1 2	1 2

NO.	QUESTIONS	ANSWER AND CODING CATEGORIES	SKIP
**Questions about your last child:**			
S4	In what month and year was your last child born?<br>*Probe:*<br>What is his/ her birthday?	Date of birth<br>Day…………………………... __ __<br>Month………………………... __ __<br>Year………………… __ __ __ __<br>DK…………………………….. 99	
S5	How old is your last child?<br>*Probe:*<br>How old was your last child at his/ her last birthday?	Age (in completed years) _____ months	
S6	Does your last child have a birth certificate?<br>*Probe:* Has your last child's birth been registered with the civil authorities?	Yes……………………………… 1<br>No……………………………… 2<br>DK……………………………... 3	
**Questions about you:**			
S7	Your marital status	Never married…………………… 1<br>Married………………………… 2<br>Cohabiting/ living together…….. 3<br>Divorced…………………………. 4<br>Separated……………………….. 5<br>Widowed………………………… 6	

S8	How old are you?	Age (in completed years)…… __ __	
S9	What is the highest level of school you attended?	No school……………………… 0<br>Preschool…………………….. 1<br>Primary……………………….. 2<br>Lower Secondary……………. 3<br>Upper Secondary……………. 4<br>Post secondary non tertiary… 5<br>Tertiary Education…………… 6	
S10	Literacy level<br>*Probe:*<br>Check when she was reading the informed consent sheet.	Cannot read at all……………… 1<br>Able to read only parts<br>of sentence………………… 2<br>Able to read the whole sentence 3	
S11	What is your occupation	Government officer/ employee.. 1<br>Merchant…………….……… 2<br>Housewife……………………… 3<br>Farmer…………….………….. 4<br>Other (specify)____________ 9	
S12	Are you a member of “Women Union”?	Yes……………………………. 1<br>No………………………………. 2	
**Questions about your husband:**			
S13	How old is your husband?	Age (in completed years)…… __ __	
S14	What is the highest level of school your <u>husband</u> attended?	No school……………………… 0<br>Preschool…………………….. 1<br>Primary……………………….. 2<br>Lower Secondary……………. 3<br>Upper Secondary……………. 4<br>Post secondary non tertiary… 5<br>Tertiary Education…………… 6	
S15	What is your <u>husband’s</u> occupation?	Government officer/ employee.. 1<br>Vender/ merchant……………. 2<br>Farmer………………………….. 3<br>No occupation………………… 4<br>Other (specify)____________ 9	
**Questions about your household:**			
S16	What is the religion of the head of this household?	Buddhist………………………... 1<br>Christianity……………………... 2<br>Animist…………………………... 3<br>Islam…………………………….. 4<br>No religion………………………. 6<br>Other religion (specify)_______ 7	
S17	To what ethnic group does the head of this household belong?	Lao……………………………… 1<br>Phutai…………………………… 2<br>Makong…………………………. 3<br>Others(specify)_____________ 9	
S18	Does your household have:<br>[A] Electricity?<br>[B] Radio?<br>[C] Television?<br>[D] Non-mobile telephone?<br>[E] Refrigerator?<br>[F] Clock?<br>[G] Fan?<br>[H] Sofa /Wooden settee?<br>[I] Water pump?<br>[J] Air conditioner?<br>[K] Washing machine?<br>[L] CD/ DVD player?<br>[M] Livestock?	Yes No<br>Electricity……………………… 1 2<br>Radio…………………………… 1 2<br>Television……………………… 1 2<br>Non-mobile telephone……..…. 1 2<br>Refrigerator…………………….. 1 2<br>Clock…………………………… 1 2<br>Fan……………………………… 1 2<br>Sofa /Wooden settee………….. 1 2<br>Water pump…………………….. 1 2<br>Air conditioner………………….. 1 2<br>Washing machine……………… 1 2<br>CD/ DVD player………………... 1 2<br>Livestock……………………… 1 2	

S19	Does any member of your household own:<br>[A] Watch<br>[B] Mobile phone<br>[C] Bicycle<br>[D] Motorcycle/ Scooter<br>[E] Animal drawn-cart<br>[F] Car/ Truck<br>[G] Boat with motor<br>[H] Tuk tuk<br>[I] Tak tak<br>[J] Camera<br>[K] Computer	Yes No<br>Watch……………………………. 1 2<br>Mobile phone…………………… 1 2<br>Bicycle………………………...… 1 2<br>Motorcycle/ Scooter……………. 1 2<br>Animal drawn-cart…………….. 1 2<br>Car/ Truck……………………… 1 2<br>Boat with motor………………… 1 2<br>Tuk tuk…………………………… 1 2<br>Tak tak…………………………… 1 2<br>Camera………………………….. 1 2<br>Computer………………………... 1 2	
S20	What is the MAIN source of water?	Piped water…………………… 1<br>Borehole………………………….. 2<br>Dug well protected……………… 3<br>Dug well not protected…………... 4<br>Water from spring……………….. 5<br>Rainwater collection…………….. 6<br>Tanker-truck………………………<br>Surface water (river, stream, 7<br>lake, dam, pond, canal)………. 8<br>Bottled water…………………….. 9<br>Other (specify)_____________ 10	
S21	What kind of toilet facility do members of your household usually use?	Flush toilet………………………... 1<br>Pit latrine………………………….. 2<br>Compositing toilet……………….. 3<br>Bucket…………………………….. 4<br>Hanging toilet…………………….. 5<br>No facility, Bush, Field…………... 6<br>Other (specify)_____________ 9	
S22	How often do you read a newspaper or magazine?<br>*(read out the choices)*	Almost every day……………….. 1<br>At least once a week…………… 2<br>Less than once a week……….. 3<br>Not at all……………………… 4	
S23	Do you listen to the radio?<br>*(read out the choices)*	Almost every day……………….. 1<br>At least once a week…………… 2<br>Less than once a week………... 3<br>Not at all……………………… 4	
S24	How often do you watch television?<br>*(read out the choices)*	Almost every day……………….. 1<br>At least once a week…………… 2<br>Less than once a week……….. 3<br>Not at all……………………… 4	
S25	During the last month, how often did you use the internet?<br>*(read out the choices)*	Almost every day……………….. 1<br>At least once a week…………… 2<br>Less than once a week……….. 3<br>Not at all……………………… 4	
S26	What is your household's income per year?	____________ kips/year	

Section II: History of Pregnancy and Delivery

Now, I would like to ask you questions about your history of your pregnancy and delivery.

NO.	QUESTIONS	ANSWER AND CODING CATEGORIES	SKIP
H1	Please tell me your age at first pregnancy.	__ __years	
H2	How many times did you become pregnant?<br>*(including miscarriage / abortion)*	____ times	
H3	How many times did you give live birth?	____ times	
H4	Have you ever had experiences with abortion?	Yes (specify)………____times 1<br>No……………………………. 2	
H5	Have you ever had still birth?	Yes (specify)………____times 1<br>No……………………………. 2	
H6	Have you ever had caesarean section?	Yes…………………………….. 1<br>No……………………………… 2	

H7	When you got pregnant with your last child, did you intended to get pregnant?	Yes…………………………….. 1<br>No……………………………… 2	1⇒H9
H8	Why because you did not want baby at that time or want no more children?	At that time, I did not want the baby………………………….. 1<br>I did not want to have baby any more……………………. 2	
H9	Did you use any contraception for birth spacing before you got pregnant with your last child?	Yes…………………………….. 1<br>No……………………………… 2	

Section III: Mother and Child Healthcare Services Utilization

I would like to ask questions about mother and child healthcare services utilization

NO.	QUESTIONS	ANSWER AND CODING CATEGORIES	SKIP
Antenatal Care			
M1	Did you see anyone for antenatal care during your pregnancy with your last child?	Yes…………………………... 1<br>No……………………………… 2	2⇒M12
M2	Did you pay for ANC visit?<br><br>(Circle all that apply)	Yes for the service…………… 1<br>Yes for the drug/medicine……. 2<br>Yes for the lab tests………….. 3<br>No payment…………………… 4	
M3	Where did you have antenatal care?<br><br>(Choose the main one you used)	Public sector<br>Central hospital………….……. 1<br>Provincial hospital………….. 2<br>District hospital………………. 3<br>Health center…………………. 4<br>Outreach………………………. 5<br>Other public (specify)_______ 6<br>Private Medical Sector<br>Private hospital………………. 7<br>Private clinic…………………... 8<br>Private maternity home……… 9<br>Other private medical (specify)_______ 10<br>Other (specify)___________ 11	
M4	What were reasons you chose this person/ place for antenatal care?<br>*Probe:*<br>Any other reasons?<br><br>(Circle all that apply)	Cheap/ lack of money………… 1<br>Nearest to home……………….. 2<br>Received antenatal care in the past………………………. 3<br>Know the person……………….. 4<br>Health staff's advice…………. 5<br>Family, relative, friend's advice. 6<br>Staff is kind, friendly…………. 7<br>Enough equipment…………….. 8<br>Had no other choice…………. 9<br>Other(specify)_____________ 10	
M5	How far is your house to this health facility?	____________ meters	
M6	What form of transport did you use to get to health facility?<br><br>(Choose the main one)	Walked………………………… 1<br>Bicycle………………………… 2<br>Motorcycle……………………. 3<br>Public bus…………………….. 4<br>Car…………………………….. 5<br>Tak tak………………………… 6<br>Others (specify)__________ 9	
M7	How long does it take you to reach to this health facility?	____________ minutes	
M8	Whom did you see for antenatal care?<br>*Probe:*<br>Anyone else?<br>*Probe for the type of person seen and circle all answers given.*	Health professional:<br>Health professional……….. 1<br>(Doctor,Nurse/Midwife,Auxiliary nurse<br>Other person<br>Traditional birth attendant….. 2<br>Community health worker….. 3<br>Other(specify)_____________ 4	

M9	How many months pregnant were you when you first received antenatal care for this pregnancy?	Months………………… __ __ months<br>DK……………………………….. 99	
M10	How many times did you receive antenatal care during this pregnancy?	Number of times…….…… __ __ times<br>DK……………………..…… 98<br>…	
M11	What actually did she/he do or did you received?<br>*Probe:*<br>Any other things?<br><br>*(Circle all that apply)*	Blood pressure………………… 1<br>Weight……………………..… 2<br>Blood test……………………….. 3<br>Urine test……………………… 4<br>Stethoscope…………………… 5<br>Doppler………………………….. 6<br>Ultrasound……………………... 7<br>Prescribe de-worming……….. 8<br>Prescribe iron………………….. 9<br>Other(specify)____________ 10	⇒M13
M12	Why did you not receive antenatal care?<br><br>*Probe:*<br>Circle all that apply.	I did not think antenatal care is important…………….…… 1<br>I did not have time……………… 2<br>Health facility is too far……….. 3<br>Money consuming…………… 4<br>Family member's decision…….. 5;who<br>Others (specify)__________ 9	
**Tetanus Toxoid Vaccination for Mothers**			
M13	Do you have a card or other document with your own immunizations listed?<br><br>*May I see it please?*<br>*If a card is presented, use it to assist with answers to the following questions.*	Yes (card seen)……………..... 1<br>Yes (card not seen)…………... 2<br>No……………………………… 3<br><br>DK……………………………… 8	
M14	When you were pregnant with your last child, did you receive any injection in the arm or shoulder to prevent the baby from getting tetanus, that is convulsions after birth?	Yes……………………………… 1<br>No………………………………. 2<br><br>DK………………………………. 8	 2⇒M16<br><br>8⇒M16
M15	How many times did you receive this tetanus injection during your pregnancy with your last child?	Number of times……………… __<br><br>DK………………………………. 8	⇒M17<br><br>8⇒M17
M16	Why did you not receive tetanus injection during pregnancy?<br><br>*Probe:*<br>Circle all that apply.	I already received 5 times…… 1<br>I did not think tetanus injection is important…………….…… 2<br>I did not have time……………… 3<br>Health facility is too far……….. 4<br>No money……….…………… 5<br>Family member's decision…….. 6;who<br>Others (specify)__________ 9	1⇒M19
M17	Did you receive any tetanus injection at any time before your pregnancy with your last child, either to protect yourself or another baby?	Yes……………………………… 1<br>No………………………………. 2<br>DK………………………………. 8	 2⇒M19<br>8⇒M19
M18	How many times did you receive a tetanus injection before your pregnancy with your last child?	Number of times……………… __<br><br>DK………………………………. 8	
M19	During this pregnancy, were you given or did you buy any iron tablets?	Yes……………………………… 1<br>No………………………………. 2<br>DK………………………………. 8	

Delivery			
M20	Where did you give birth to your last child?<br>*Probe to identify the type of source.*<br><br>*If unable to determine whether public or private, write the name of the place.*<br><br>______________________<br>*(Name of place)*	Home<br>Your home…………………….. 1<br>Other home…………………… 2<br>Public sector<br>Central hospital……….……… 3<br>Provincial hospital………….. 4<br>District hospital………………. 5<br>Health center…………………. 6<br>Other public (specify)_______ 7<br>Private Medical Sector<br>Private hospital………………. 8<br>Private clinic………………….. 9<br>Private maternity home……… 10<br>Other private<br>medical (specify)________ 11<br>Other (specify)____________ 19	 1⇒M28<br>2⇒M28
M21	Did you pay for the delivery?<br><br>Circle all that apply.	Yes for services………………… 1<br>Yes for drug/medication………. 2<br>Yes, other(specify)__________ 3<br>No payment……………………. 4	 <br><br>4⇒M23
M22	What was the total expenditure for the delivery per time?	____________________ kip	
M23	How far is your house to this health facility?	____________ meters	
M24	What form of transport did you use to get to health facility?	Walked……………………….. 1<br>Bicycle……………………….. 2<br>Motorcycle……………………. 3<br>Public bus…………………….. 4<br>Car…………………………….. 5<br>Tak tak……………………….. 6<br>Others (specify)__________ 9	
M25	How long does it take you to reach to this health facility?	____________ minutes	
M26	(Answer only if you delivered in a health facility.)<br>How long did you stay there after the delivery?<br><br>If less than one day, record hours. If less than one week, record days. Otherwise, record weeks.	 Hours…………….__ __ hours 1<br>Days……………..__ __ days<br>Weeks…………__ __ weeks 2<br><br>Don't know/ remember……….. 3<br><br>8	
M27	What were reasons you chose this person/ place for assisting the delivery?<br><br>*Probe:*<br>Any other reasons? (Answer all that apply)	Cheap/ lack of money………… 1<br>Nearest from home…………….. 2<br>Gave delivery in the past…….. 3<br>Receiving antenatal care……… 4<br>Know the person……………….. 5<br>Health staff's advice………….. 6<br>Family, relative, friend's advice. 7⇒<br>Staff is kind, friendly………….. 8<br>Enough equipment…………….. 9<br>Safety…………………………… 10<br>Had no other choice………….. 11<br>Other(specify)_____________ 96	 <br><br><br><br><br>Who__
M28	Who assisted with the delivery of your last child?<br>*Probe:*<br>Anyone else?<br>*Probe for the type of person assisting and circle all answers given.*<br>*If respondent says no one assisted, probe to determine whether any adults were present at the delivery.*	Health professional:<br>Health professional………….. 1<br>Other person<br>Traditional birth attendant…… 2<br>Community health worker…… 3<br>Husband………………………. 4<br>Relative / Friend……………… 5<br>Other(specify)_____________ 6<br>No one………………………….. 7	 1⇒M30

M29	Ask ONLY to those who was NOT assisted by health professional or to whom home delivery.<br><br>What were reasons you did not ask health professional to assist you in the delivery?<br>*Probe:*<br>Any other reasons?<br><br>(Answer all that apply)<br><br><br>Health professional/ Skilled birth attendant:<br>Doctor, Nurse, Midwife	Delivery was too fast………… 1<br>Expensive/ no money………….. 2<br>Health facility is too far……….. 3<br>No means of transportation…… 4<br>Waiting time is too long……….. 5<br>Delivery was easy and no need to ask for professional……… 6<br>Other children was born the same……………………….... 7<br>Don't know where to go………. 8<br>Prefer TBA, Relative………….. 9<br>No one to look after the house/ children……………… 10<br>Health staff's advice………….. 11<br>Family, relative, friend's advice. 12⇒<br>Not friendly…………………….. 13<br>Service providers are male…… 14<br>Other(specify)_____________ 96	Who_
M30	Did you have any problems during the delivery with your last child?	Yes………………………….. 1<br>No…………………………… 2<br>DK……………………………. 8	
M31	Did the last child have any health problem?	Yes……………………………. 1<br>No…………………………… 2<br>DK……………………………. 8	
M32	How much did your last child weigh?<br>*Record weight from health card, if available.*	From card……__________g 1<br>From recall……__________g 2<br>Did not weighed…………….. 3<br>DK……………………………. 8	
**Postnatal Health Checks**			
M33	After the delivery, did anyone check on YOUR health?	Yes………………………….. 1<br>No…………………………..… 2	2⇒M36
M34	Where did this check take place?	Home<br>Your home…………………….. 1<br>Other home…………………… 2<br>Public sector<br>Central hospital……….……… 3<br>Provincial hospital………….. 4<br>District hospital……………… 5<br>Health center………………… 6<br>Other public (specify)_______ 7<br>Private Medical Sector<br>Private hospital……………… 8<br>Private clinic………………….. 9<br>Private maternity home……… 10<br>Other private medical (specify)_______ 11<br>Other (specify)____________ 19	
M35	What actually did she/he do?<br>*Probe:*<br>Any other things?<br><br>*(Circle all that apply)*	Clean the wound…………….. 1<br>Got injection………………….. 2<br>Examine uterus contraction…… 3<br>Examine/ take care of breast…. 4<br>Teach how to breastfeed……… 5<br>Advise how to take care of baby 6<br>Advise how to take care of mother herself………….. 7<br>Examine baby's condition…….. 8<br>Bathe the baby……………….. 9<br>Other(specify)_____________ 10	
M36	Did anyone check on your last child's health after the delivery?<br>(Ask regardless health facility or home delivery)<br>-Example: someone examine the baby's cord, or check the baby is okay	Yes………………………..…….. 1<br>No……………………………. 2	2⇒M39

M37	Where did this check take place?	Home<br>Your home…………………….. 1<br>Other home…………………… 2<br>Public sector<br>Central hospital……….……… 3<br>Provincial hospital………….. 4<br>District hospital………………. 5<br>Health center…………………. 6<br>Other public (specify)_______ 7<br>Private Medical Sector<br>Private hospital………………. 8<br>Private clinic…………………... 9<br>Private maternity home……… 10<br>Other private<br>medical (specify)________ 11<br>Other (specify)____________ 19	
M38	Who checked on your last child's health at that time?	Health professional<br>Health professional………….. 1<br>Other person<br>Traditional birth attendant……. 2<br>Community health worker……. 3<br>Relative/ Friend……………….. 4<br>Other (specify)____________ 5	
M39	How soon after birth did you first bath your first child?	Immediately after birth………… 1<br>Within 24 hours of birth……… 2<br>After 24 hours of birth………….. 3<br>DK……………………………….. 8	
**Breastfeeding Practice**			
M40	Did you ever breastfeed your last child?	Yes………………………………. 1<br>No…………………………….…. 2	2⇒M42
M41	How long after birth did you first put your last child to the breast?<br>*If less than 1 hour, record '00' hours. If less than 24 hours, record hours. Otherwise, record days.*	Immediately…………………….. 1<br>Hours……………………__ __ 2<br>Days……………….….__ __ 3<br>Don't know/ remember……….. 8	1⇒M43<br>2⇒M43<br>3⇒M43<br>8⇒M43
M42	Why did you not breastfeed?<br><br>Skip if you breastfeed your last child:	Insufficient……………………… 1<br>No time………………………….. 2<br>Baby did not suck/ drink……. 3<br>Breastmilk is not good for<br>Health………………………… 4<br>Others(specify)………………… 9	
M43	Did you exclusively breastfeed your last child for 6 months?	Yes………………………….. 1<br>No……………………………… 2	1⇒M46<br>2⇒M44
M44	In the first three days after delivery, was your last child given anything to drink/eat other than beast milk?	Yes………………………………. 1<br>No…………………………….… 2	2⇒M46
M45	What was your last child given to drink or food?<br><br>*Probe:*<br>Anything else?<br><br>*(Circle all that apply)*	Rice………………………….. 1<br>Rice-powdered(Cerelac)…….. 2<br>Milk (other than breast milk)…... 3<br>Plain water…………………….. 4<br>Sugar or glucose water……….. 5<br>Gripe water…………………… 6<br>Sugar-salt-water solution……… 7<br>Fruit juice……………………….. 8<br>Infant formula………………….. 9<br>Tea / Infusions………………….. 10<br>Honey……………………….…. 11<br>Other (specify)____________ 12	
M46	Do you still breastfeed your last child?	Yes……………………….….….. 1<br>No…………………………..…… 2	

Family Planning/ Contraceptive Methods

M47	Are you using any contraceptive methods?	Yes………………………………. 1<br>No……………………………… 2	2⇒M50
M48	What kind of contraceptive method do you use?<br><br>*Do not prompt.*<br>*If more than one method is mentioned, circle all that apply.*	Female sterilization…………… 1<br>Male sterilization……………….. 2<br>IUD……………………………… 3<br>Injectables……………………… 4<br>Implants………………………… 5<br>Pill……………………………….. 6<br>Male condom…………………… 7<br>Female condom……………….. 8<br>Diaphragm……………………... 9<br>Foam / Jelly…………………….. 10<br>Lactational amenorrhoea Method (LAM)……………….. 11<br>Periodic abstinence / Rhythm… 12<br>Withdrawal……………………… 13<br><br>Other (specify)____________ 15	 <br><br><br><br><br><br><br><br><br><br>11⇒M50<br>12⇒M50<br>13⇒M50<br><br>15⇒M50
M49	Where did you obtain (current method) the last time?<br><br>*Probe to identify the type of source. If unable to determine if public or private sector, write the name of the place.*<br><br>____________________<br>*(name of the place)*	Public Sector<br>Central Hospital………………. 1<br>Provincial Hospital……………. 2<br>District Hospital………………. 3<br>Health Center…………………. 4<br>Lao Youth Center LYC……….. 5<br>Outreach Team……………….. 6<br>Village Health Volunteer VHV.. 7<br>Other Public Sector_______ 8<br><br>Private Medical Sector<br>Private Clinic………………….. 9<br>Pharmacy……………………… 10<br>Private Doctor………………… 11<br>Other Private Medical Sector 12<br>(specify)______________<br><br>Other Source<br>Shop…………………………. 13<br>Friend/ Relative…………….. 14<br>Other (specify)___________ 15	Go to Next Module for all responses

Immunization

M50	Did your last child receive the vaccination?<br>*If yes:*<br>May I see it and take photos?	Yes, with card………………….. 1<br>Yes, without card……………… 2<br>No, did not receive the vaccination…………………… 3	 2⇒M52,EPI<br><br>3⇒M56

M51	a) Copy dates for each vaccination from the card.<br>b) Write '99' in day column if card shows that vaccination was given but no date recorded.	Date of Immunization			
		Day	Month	Year	
	BCG				
	HepB0 at birth				
	DPT-HepB-Hib/Polio/PCV 1				
	DPT-HepB-Hib/Polio/PCV 2				
	DPT-HepB-Hib/Polio/PCV 3				
	Measles and Rubella (MR)				
	Japanese Encephalitis Vaccine				
	Vitamin A (most recent)				
	ABZ/MBZ (most recent)				

M52	Where did your last child's last vaccination take place?	Public Sector<br>Central Hospital………………… 1<br>Provincial Hospital…………….. 2<br>District Hospital……………… 3<br>Health Center…………………. 4<br>Lao Youth Center LYC………. 5<br>Outreach Team……………….. 6<br>Village Health Volunteer VHV.. 7<br>Other Public Sector_______ 8<br><br>Private Medical Sector<br>Private clinic………………… 9<br>Pharmacy……………………… 10<br>Private Doctor………………… 11<br>Other Private Medical Sector<br>(specify)______________ 12<br><br>Other(specify)____________ 13	
M53	How far is your house to this health facility?	___________ meters	
M54	What form of transport did you use to get to health facility?	Walked………………………… 1<br>Bicycle………………………… 2<br>Motorcycle…………………… 3<br>Public bus…………………….. 4<br>Car…………………………….. 5<br>Tak tak………………………… 6<br>Others (specify)_________ 9	
M55	How long does it take you to reach to this health facility?	___________ minutes	⇒M57
M56	If mother has not received immunization, ask:<br>Why did your last child did NOT get immunizations?<br><br>(Circle all that apply)	Lack of time…………………….. 1<br>Not considered important..…… 2<br>Forgot…………………………… 3<br>Too far to health facility……… 4<br>Fear to side-effect……………. 5<br>Traditional restriction/belief…… 6<br>Family member's advice……… 7<br>Did not know when/where to go 8<br>Other(specify)____________ 9	
M57	What did you practice from pregnancy to child bearing about any traditional practice or belief?<br><br>(Circle all that apply)	Yu fai……….…………. 1<br>Certain foods restriction.. 2<br>Bathing newborn………. 3<br>Discard colostrums…….. 4<br>Keep baby without clothes.. 5<br>Restriction on contact with people…………..…. 6<br>No did not practice any traditional or belief………….. 7<br>Others(Specify)_________ 9	

		A. Husband		B. Other family member/Relatives		
M58	Have you ever discuss the following maternal and child health services with your husband and/or other family member/relatives?	Yes	No	Yes	No	
	ANC	1	2	1	2	
	Tetanus toxiod injection	1	2	1	2	
	Delivery place	1	2	1	2	
	Delivery assistant	1	2	1	2	
	PNC	1	2	1	2	
	Contraceptive methods use	1	2	1	2	
	Immunization	1	2	1	2	

Section IV: Knowledge on MNCH

Now I would like to ask you questions about your knowledge on MNCH

NO.	QUESTIONS	ANSWER AND CODING CATEGORIES		SKIP
**Antenatal Care**				
K1	Do you know what the purposes of “antenatal care”?<br><br>(Circle all that apply)	To check the baby’s health……..<br>Pregnancy monitoring…………<br>Risk in pregnancy assessing….<br>Sexually transmitted<br>Infections screening…………<br>Health education……………..<br>No I do not know………………<br>Others(specify) ____________________	1<br>2<br>3<br><br>4<br>5<br>6<br>9	
K2	Do you know at least how many times of antenatal check visits are recommended?	Yes…………………….___times<br>No………………………………	1<br>2	
K3	During which months of pregnancy pregnant woman should first receive antenatal care?	<4 months……………………<br><6 months……………………<br>>6 months……………………...<br>DK………………………………	1<br>2<br>3<br>4	
K4	Have you ever heard about the risk factors of pregnancy?	Yes………………………………<br>No…………………………………	1<br>2	 2⇒K6
K5	What are they (the risks for pregnancy)?<br><br>*Probe:*<br>Let mother speak out, don’t prompt mother<br><br>(Circle only what mother mentioned, multiple answer possible)	Mother age ≦19 years old……..<br>Mother age ≧35years old………<br>Ever stillbirth……………………<br>Ever abortion……………………<br>Prior baby birth weight <2500g…<br>Experience of caesarean section<br>Severe blood loss during<br>Previous pregnancy…………..<br>Experience of manual or scrape<br>to remove placenta……………<br>Mother height <145cm…………<br>Others (specify)____________	01<br>02<br>03<br>04<br>05<br>06<br><br>07<br><br>08<br>09<br>10	
K6	Do you know, in total, how many kilograms a pregnancy needs to gain for mature pregnancy?	< 10 kg……………………………<br>10 – 12 kg………………………..<br>> 12kg……………………………..<br>DK…………………………………	1<br>2<br>3<br>4	
K7	Do you know tetanus toxoid injection is recommended especially for pregnant woman?	Yes…………………………………<br>No………………………………….	1<br>2	
K8	Do you know the use of insecticide treated bed nets from prenatal to postnatal is recommended?	Yes…………………………………<br>No………………………………….	1<br>2	
K9	Do you know taking iron is recommended during pregnancy?<br>Show the iron tablet	Yes…………………………………<br>No………………………………….	1<br>2	

K10	What are some danger or warning signs during pregnancy?<br>*Probe*: Don't prompt mother, let her speak out and listen carefully.<br><br>(Circle only what mother mentioned, multiple answer possible)	Stillbirth……………………………..<br>Feeling excessive weakness……<br>Light bleeding / Spotting…………<br>Haemorrhage / Heavy bleeding...<br>Severe abdominal pain…………..<br>Convulsion/ Fits…..………………<br>High blood pressure..……………<br>Swelling on hands/ face…………<br>Severe headache………………...<br>Blurred eye vision………………...<br>Fever/ Sepsis……………………..<br>Foul Smelling discharge…………<br>Anemia/ Pallor……………………<br>Cessation of fetal movement /<br>Baby does not move…………<br>Abnormal lie / Position of fetus….<br>Multiple pregnancy /<br>Large abdomen………………..<br>Obstructed / Prolonged /<br>Sunset two times……………..<br>Other(specify)_____________<br>Don't know………………………..	01<br>02<br>03<br>04<br>05<br>06<br>07<br>08<br>09<br>10<br>11<br>12<br>13<br><br>14<br>15<br><br>16<br><br>17<br>96<br>98	
**Delivery**				
K11	What are some danger or warning signs of a problem with delivery?<br>*Probe*: Don't prompt mother, let her speak out and listen carefully.<br>(Circle only what mother mentioned, multiple answer possible)	Severe bleeding…………………<br>Prolonged labor/ Difficult labor….<br>Convulsion/ Preeclampsia...…….<br>Perineal laceration/ Tear………..<br>Fever……………………………..<br>Green or brown waters…………<br>Umbilical cord prolapsed………..<br>Retained placenta………………<br>Other(specify)_____________	1<br>2<br>3<br>4<br>5<br>6<br>7<br>8<br>9	
K12	Do you know the appropriate place for delivery?	Yes……………………………….<br>No……………………………….	1<br>2	 2⇒K14
K13	If "yes", which place was suggested to give delivery?	Government hospital…………<br>Health center……………………<br>Home…………………………….<br>Other (specify)____________	1<br>2<br>3<br>9	
**Postnatal Health Checks**				
K14	What are some danger or warning signs of a problem with newborn baby?<br>*Probe*: Don't prompt mother, let her speak out and listen carefully.<br>(Circle only what mother mentioned, multiple answer possible)	Red spot/rush……………………..<br>Low birth weight/ Small size…….<br>Born prematurely/ Too early……..<br>Poor feeding or sucking…………<br>Fast breathing…………………….<br>Keep vomiting…………………….<br>Baby is too warm or too cold……<br>Eyes with pus discharge…………<br>Cord with redness or pus………..<br>High fever…………………………<br>Baby looking yellowish/Jaundice.<br>Unresponsive/ Weak cry…………<br>Other(specify)_____________<br>Don't know………………………..	01<br>02<br>03<br>04<br>05<br>06<br>07<br>08<br>09<br>10<br>11<br>12<br>19<br>98	
K15	What are some danger or warning signs that can occur to the mothers during the first 2 days after the delivery?<br><br>(Circle only what mother mentioned, multiple answer possible)	Feeling excessive weakness……<br>Haemorrhage / Heavy bleeding...<br>Severe abdominal pain…………..<br>Convulsion/ Fits…..………………<br>Swelling on hands/ face…………<br>Severe headache………………...<br>Blurred eye vision………………...<br>Fever/ Sepsis……………………..<br>Foul Smelling discharge…………<br>Difficulty breathing………………<br>Other(specify)_____________<br>Don't know………………………..	01<br>02<br>03<br>04<br>05<br>06<br>07<br>08<br>09<br>10<br>19<br>98	

K16	How long after birth should a newborn baby bathed?	Immediately after birth………….. 1<br>Within 24 hours of birth………… 2<br>After 24 hours of birth…………… 3<br>Other(specify)______________ 4<br>Don't know……………………….. 8	
**Breastfeeding Practice**			
K17	Do you know the benefits of breastmilk/ breast feeding?<br>Don't prompt mother, let her speak out and listen carefully.<br>(Circle only what mother mentioned, multiple answer possible)	Good for child's health…………… 1<br>Contains immunities to diseases. 2<br>Easily digestible………………….. 3<br>Enhances brain………………… 4<br>Promotes bonding between mother and baby………………… 5<br>Provide perfect infant nutrition…. 6<br>Helps shrink mother's uterus…… 7<br>Don't know………………………… 8<br>Other(specify)______________ 9	
K18	How long after birth should a newborn baby first breastfeed?	Immediately after birth…………... 1<br>Within an hour of birth…………… 2<br>After one hour……………………. 3<br>Other(specify)______________ 4<br>Don't know……………………….. 8	
K19	Do you think breastfeeding alone provides sufficient nutrition in the first few months after the birth?	Yes………………………………… 1<br>No…………………………………. 2	
**Family Planning/ Contraceptive Methods**			
K20	Have you ever heard of methods for contraceptive methods to delay or avoid a pregnancy?<br><br>(Circle only what mother mentioned, multiple answer possible)	Female sterilization…………….. 01<br>Male sterilization……………….. 02<br>IUD……………………………… 03<br>Injectables……………………… 04<br>Implants………………………… 05<br>Pill……………………………….. 06<br>Male condom…………………… 07<br>Female condom………………… 08<br>Diaphragm……………………… 09<br>Foam / Jelly…………………….. 10<br>Lactational amenorrhoea Method (LAM)……………….. 11<br>Periodic abstinence / Rhythm… 12<br>Withdrawal……………………… 13<br>Do not know any methods……… 14<br>Other (specify)____________ 15	
K21	Do you know a place where you can obtain a method of family planning?	Yes……………………………….. 1<br>No………………………………… 2	2⇒K23
K22	Where is that?<br><br>Any other place?<br>*Probe to identify the type of source.*<br><br>(Circle only what mother mentioned, multiple answer possible)	Public Sector<br>Central Hospital……………… 1<br>Provincial Hospital…………… 2<br>District Hospital……………… 3<br>Health Center………………… 4<br>Lao Youth Center LYC………. 5<br>Outreach Team………………... 6<br>Village Health Volunteer VHV.. 7<br>Other Public Sector_______ 8<br><br>Private Medical Sector<br>Private hospital/ Clinic………. 9<br>Pharmacy……………………… 10<br>Private Doctor………………… 11<br>Mobile Clinic…………………… 12<br>Field Worker…………………… 13<br>Other Private Medical Sector (specify)______________ 14<br>Other Source<br>Shop……………………………. 15<br>Friend/ Relative………………. 16<br>Other (specify)___________ 19	

**Immunization**			
K23	Do you think that vaccination can prevent from some diseases?	Yes…………………………………… 1<br>No……………………………………. 2	
K24	Do you know that vaccination is not just for children but for all ages?	Yes…………………………………… 1<br>No……………………………………. 2	
K25	Do you know what kind of child's health conditions you should not bring your child to get vaccination?	Yes…………………………………… 1<br>No……………………………………. 2	2⇒K27
K26	What kind of health condition is that?<br>(Circle only what mother mentioned, multiple answer possible)	When child has fever or sick..………… 1<br>When child had a life-threatening allergic reaction before………………………… 2<br>When parents had experience of life-threatening allergic reaction before 3<br>When child is taking high-dose corticosteroids………………………….. 4<br>When child has with weakened immune system………………………………… 5<br>Others(specify)____________________ 9	
**Continuum of Care**			
K27	Have you ever heard about the free maternal and child care health services provided by the Government of Laos?	Yes…………………………………… 1<br>No……………………………………. 2	
K28	Do you know the importance of continuously receiving health care from pregnancy to post delivery?	Yes…………………………………… 1<br>No……………………………………. 2	
K29	Who did you receive health information or message the most to each service?<br>Health professional<br>1.Health professional<br>Other person<br>2.Traditional birth attendant<br>3.Community health worker<br>4.Village head<br>5.Husband<br>6.Relative/ Friend<br>Other Source<br>7.Shop<br>8.Radio<br>9.Newspaper<br>10.TV<br>11.Other (specify)	ANC………………………………. ___<br>Tetanus toxoid injection……….. ___<br>Delivery place…………………… ___<br>Delivery assistant……………….. ___<br>Postnatal care…………………… ___<br>Family planning………………….. ___<br>Immunization…………………… ___	

END OF SURVEY
THANK YOU VERY MUCH FOR YOUR COOPERATION
